# Promoter Specificity and Efficacy in Conditional and Inducible Transgenic Targeting of Lung Macrophages

**DOI:** 10.3389/fimmu.2017.01618

**Published:** 2017-11-24

**Authors:** Alexandra L. McCubbrey, Kristen C. Allison, Alisa B. Lee-Sherick, Claudia V. Jakubzick, William J. Janssen

**Affiliations:** ^1^Department of Medicine, National Jewish Health, Denver, CO, United States; ^2^Division of Critical Care Medicine and Pulmonary Sciences, University of Colorado Denver, Denver, CO, United States; ^3^Department of Pediatrics, University of Colorado Anschutz Medical Campus, Aurora, CO, United States; ^4^Department of Pediatrics, National Jewish Health, Denver, CO, United States

**Keywords:** macrophage, Cre, lung, conditional, LysM-Cre, CX3CR1-estrogen receptor-Cre, CD68-rtTA, Csf1r-Cre

## Abstract

Conditional and inducible Cre-loxP systems are used to target gene deletion to specific cell lineages and tissues through promoter-restricted expression of the bacterial DNA recombinase, Cre. Although Cre-loxP systems are widely used to target gene deletion in lung macrophages, limited data are published on the specificity and efficiency of “macrophage targeting” Cre lines. Using R26-stop^fl/fl^-TdTomato and tetOn-GFP reporter lines, we assessed the specificity and efficiency of four commercially available Cre driver lines that are often considered “macrophage specific.” We evaluated two conditional (Csf1r-Cre and LysM-Cre) and two inducible [CX_3_CR1-estrogen receptor-Cre (ERCre) and CD68-rtTA] lines. We assessed Cre activation in six resident lung myeloid populations, as well as activation in lung leukocytes, lung epithelial and endothelial cells, peripheral blood leukocytes, and tissue macrophages of the spleen, bone marrow, and peritoneal cavity. Although Csf1r-Cre and LysM-Cre target resident alveolar macrophages (ResAM) and interstitial macrophages (IM) with high efficiency, neither line is specific for macrophages. Csf1r-Cre targets all leukocyte populations, while LysM-Cre targets dendritic cell, neutrophils, monocytes, and a quarter of lung epithelial cells. CX_3_CR1-ERCre and CD68-rtTA both target IM, but do not target ResAM. Further, although neither line is specific for macrophages, a pulse-wait administration of tamoxifen or doxycycline can be used to significantly improve IM specificity in these inducible lines. In summary, while Cre-loxP remains a powerful tool to study macrophage function, numerous pitfalls exist. Herein, we document strengths and weaknesses of Csf1r-Cre, LysM-Cre, CX_3_CR1-ERCre, and CD68-rtTA systems for targeting specific macrophage populations in the lungs and provide data that will aid investigators in selecting the proper strain.

## Introduction

*In vivo* studies are critical for understanding the homeostatic functions of lung macrophages and their roles in pulmonary disease. In this context, conditional transgenic targeting of cell populations using a Cre-loxP strategy is one of the most powerful tools available for *in vivo* studies of macrophages. However, recent work has exposed the limited macrophage specificity of many “macrophage targeting” Cre driver lines (1–3). Accurate data interpretation requires a firm understanding of the underlying genetic principles of Cre-loxP mice and detailed knowledge regarding the limitations of the chosen Cre driver.

Cre is a bacterial recombinase that catalyzes recombination between paired DNA recognition sites termed LoxP sites (4, 5). Restricted Cre expression can be achieved through the use of a cell-specific promoter that drives expression of Cre only in the cells that activate the chosen promoter; this is termed a *conditional* transgene. When a mouse line with a conditional Cre driver is crossed with a line in which a gene of interest is “floxed” with LoxP sites, the floxed gene is deleted only in cells where Cre is expressed. As this change is genomic, it will remain through the life of the targeted cell and all daughter cells.

Conditional inducible systems represent an enhancement over standard conditional systems by enabling temporal control of transgenes. The two most common include estrogen receptor-Cre (ERCre) (6) and tetracycline-On-Cre (tet-On-Cre) (7, 8). Conditionally expressed ERCre requires tamoxifen as a co-factor for Cre activity; until tamoxifen is administered, the mouse remains, essentially, “wild-type.” Similarly, tet-On-Cre systems require the administration of doxycycline to allow a conditionally expressed rtTA to bind to a tet-On promoter and induce Cre expression. However, conditional and conditional inducible systems are only as strong as the chosen conditional promoter. It has become increasingly clear that macrophage “specific” Cre driver lines have promiscuous activation in a broad range of myeloid cells (1–3, 9).

During homeostasis, the lung contains at least six unique extravascular myeloid cell populations that are defined by their location and site of origin, and distinguished by their cell surface marker repertoires. Resident alveolar macrophages (ResAM) reside in the airspace lumen and arise during embryogenesis from fetal liver monocytes (10–12). In comparison, three subpopulations of interstitial macrophages (IM) are located in the lung tissue and are believed to replenish more readily, throughout the lifespan of the mouse, from circulating Ly6C^hi^ monocytes (13, 14). Notably both ResAM and IM are capable of self-renewal and are maintained with limited contribution from adult bone marrow-derived monocytes (11, 14). IM can be divided into subpopulations based on their expression of CD11c, CD206, and MHCII: IM1 (CD206^+^MHCII^−^), IM2 (CD206^+^MHCII^+^), and IM3 (CD206^−^CD11c^+^) (14). Dendritic cells (DCs) comprise the remaining myeloid cells in the homeostatic lung and include CD11b^+^ and CD103^+^ DC subsets (15, 16).

In the setting of inflammation, additional myeloid cells infiltrate the lung tissues, including neutrophils, eosinophils, and monocytes. The absolute number of macrophages in the lung tissue also expands (17), presumably driven by infiltrating monocytes. Further, a second macrophage population appears in the alveolus, termed recruited macrophages (RecMΦ) (18, 19). Prior research suggests that these RecMΦ differentiate from infiltrating Ly6C^hi^ monocytes (20–22). Clear surface markers have been established to separate ResAM, IM1, IM2, IM3, and RecMΦ from other myeloid cells (14, 23, 24), but functional studies of macrophages *in vivo* remain a challenge.

To determine the optimum systems for targeting specific macrophage cell populations in the lungs, we selected four commercially available “macrophage” Cre driver lines. These included two conditional lines (Csf1r-Cre and LysM-Cre) and two inducible ones (CX_3_CR1-ERCre and CD68-rtTA). While these “macrophage” targeting lines have been heavily used, the pitfalls of their use have been largely ignored, in part because a systemic analysis of their specificity in the lung has not been performed. We crossed these Cre driver lines with various fluorescent reporter lines, discussed in detail below, to assess Cre activity in lung macrophages during homeostasis and inflammation. We also examined peripheral blood, since current evidence suggests that circulating monocytes give rise to macrophage subsets during inflammation. Both LysM-Cre and Csf1r-Cre strongly targeted ResAM and IM. However, we found that Csf1r-Cre (25) non-specifically targeted all leukocytes, including lymphocytes, while LysM-Cre (26) problematically targeted neutrophils and a quarter of lung epithelial cells. Activation of CX_3_CR1-ERCre (11) and CD68-rtTA (27) was strongly observed in IM, but absent in ResAM. Therefore, while CX_3_CR1-ERCre and CD68-rtTA are promising models for the study of IM, all four systems struggle to specifically target Cre activity to ResAM.

## Methods

### Animals

This study was approved and performed in accordance with the ethical guidelines of the Institutional Animal Care and Use Committee at National Jewish Health (approval #AS2574-01-20). All mice were obtained from Jackson Laboratories (Bar Harbor, ME, USA) and bred at National Jewish Health. LysM-Cre (B6.129P2-*Lyz2tm1(cre)Ifo*/J; stock number 004781), Csf1r-cre (FVB-Tg(Csf1r-icre)1Jwp/J; stock number 021024), and CX_3_CR1-ERT2 (B6.129P2(C)-*CX_3_CR1tm2.1(cre/ERT2)Jung*/J; stock number 020940) mice were crossed with R26-stop^fl/fl^-TdTomato (B6.Cg-Gt(ROSA)26Sortm14(CAG-tdTomato)Hze/J; stock number 007914) mice. Experimental animals from these three crosses were hemizygous for both genes. hCD68-rtTA (B6.Cg-Tg(CD68-rtTA2S*M2)3Mpil/Mmjax; stock number 32044-JAX) and R26-M2rtTA (B6.Cg-Gt(ROSA)26Sortm1(rtTA*M2)Jae/J; stock number 006965) mice were crossed with tetOn-GFP (Tg(tetO-HIST1H2BJ/GFP)47Efu/J; stock number 005104) mice. Experimental animals from hCD68-rtTA x tetOn-GFP were homozygous for both genes, while R26-rtTA x tetOn-GFP were hemizygous for both genes. Both male and female mice were used in these experiments.

### Doxycycline and Tamoxifen Administration

Normal diet chow was replaced with doxycycline chow (Teklad laboratory diets, NJ, USA) for 1 week, then mice were analyzed on day 7 (pulse). Doxycycline chow was dosed at 625 mg doxycycline/kg chow, designed to provide 2–3 mg of doxycycline/day based on consumption of 4–5 g chow/day. Additional analyses were performed after mice were returned to normal diet for 1, 2, or 4 weeks (wait). Mice were allowed to eat chow *ad libitum*. Tamoxifen (Sigma) suspended in neutral oil was given at 0.25 mg/g of body weight by intraperitoneal (IP) injection. Mice were injected on day 1, again on day 4, then analyzed on day 7 (pulse), Additional analyses were performed 1, 2, or 4 weeks after the last tamoxifen injection (wait).

### Tissue Harvest

Mice were euthanized by CO_2_ inhalation or IP injection of Fatal Plus (Vortech Pharmaceuticals, Dearborn, MI, USA). Peritoneal lavage (PL), bronchoalveolar lavage (BAL), lung, blood, bone marrow, and spleen were collected on ice in the following order. PL was collected in 10 mL of PBS containing 0.5 mM EDTA, pelleted, and resuspended in HBSS containing 0.3% BSA and 0.3 mM EDTA. BAL was collected in 5 mL of PBS containing 0.5 mM EDTA, pelleted, and resuspended in HBSS containing 0.3% BSA and 0.3 mM EDTA. Blood was collected by cardiac puncture using a syringe coated with 50 µL of 100 mM EDTA, then placed directly into 10 mL of PharmLyse buffer (BD Biosciences, San Jose, CA, USA) for 20 min on ice. After 20 min, blood cells were washed and resuspended in HBSS containing 0.3% BSA and 0.3 mM EDTA. Lungs were perfused by injecting 10 mL PBS through the right ventricle, after which lung tissue was visibly blanched. Lung tissue was finely chopped with a razor blade then incubated at 37°C in 1 mL of 0.4 mg/mL Liberase TM (Roche, Indianapolis, IN, USA) in RPMI for 25 min. Following incubation, tissue was pipetted rapidly up and down to further disaggregate, then filtered through 100 µm filters. Lung cells were pelleted and residual RBC were lysed for 30 s using 1 mL of BD PharmLyse (BD Biosciences, San Jose, CA, USA). BAL, lung, blood, and PL cells were washed in HBSS and resuspended in HBSS containing 0.3% BSA and 0.3 mM EDTA. Spleen tissue was chopped and mechanically dissociated, then filtered through 100 µm filters. Bone marrow was flushed from the cavities of the femur and tibia with cold PBS and mechanically dissociated, then filtered through 100 µm filters. Residual RBC in spleen and bone marrow were lysed for 10 min using 1 mL of Gey’s Solution (0.155 M NH4Cl 10 mM KHCO3 aqueous). The hemolysis wash was repeated. Spleen and BM cells were washed and resuspended in PBS containing 2% FBS, 2 mM EDTA, and 0.09% NaN_3_.

### Flow Cytometry

Flow cytometry was performed on single cell suspensions. Cells were protected from light and incubations were performed on ice. Single cell suspensions were treated with unlabeled CD16/CD32 for 30 min to block non-specific FcyR-mediated binding. Cells were stained with surface antibody panels to identify myeloid populations for 1 h, and then washed. When necessary, cells were incubated with streptavidin-conjugated secondary antibody for another 45 min, and then washed. HBSS containing 0.3% BSA and 0.3 mM EDTA was used as a buffer for all incubations. Single cell suspensions of BAL, lung, blood, and PL were analyzed using an LSRII flow cytometer (BD) and FlowJo software (Tree Star, Ashland, OR, USA). Spleen and bone marrow cells were analyzed using a YETI cell analyzer (Propel Labs). IM1, IM2, IM3, and γδ T cells were the smallest populations examined; data collection was performed such that >400 events for each of these populations were analyzed, for most populations >5,000 events were analyzed. Primary antibodies were used in various combinations as follows (source/clone): unlabeled CD16/32 (eBioscience/93), Ly6G (BD/IA8), MHCII (BD/114.15.2), F4/80 (ebioscience/BM8), CD45 (BD/30-F11), Ly6C (eBioscience/HK1.4), Siglec-F (BD/E50-2440), CD11c (eBioscience/N418), CD11b (eBioscience/M1/70), CD103 (eBioscience/2E7), CD115 (eBioscience/AFS98), CD64 (Biolegend/X54-5/7.1), MerTK (R&D/BAF591), CD3 (Biolegend/17A2), CD19 (eBioscience/eBio1D3), NK1.1 (Biolegend/PK136), γδTCR (eBioscience/eBioGL3), EpCAM (Biolegend/G8.8), CD31 (Biolegend/390), Thrombomodulin (R&D/AF3894), CD206 (Biolegend/C068C2), Tim4 (Biolegend/F31-5G3), and Gr-1 (BD/RB6-8C5). All primary antibodies were used at a 1:400 dilution except EpCAM (1:1,000), and MerTK (1:100). Streptavidin-conjugated FITC, AF647, and PE secondary antibodies (Jackson Immunolaboratories) were used at 1:200.

### LPS Injury

LPS (*Escherichia coli* 055:B5; List Biological Laboratories, Campbell, CA, USA) was administered in a dose of 20 µg in 50 µL of PBS. LPS was instilled intratracheally (IT) using a modified gavage needle. Mice were sedated with isoflurane (Baxter, Deerfield, IL, USA) before IT instillations. Reporter expression was assessed in BAL, lung, and blood 6 days after LPS instillation.

## Results

### Conditional Cre Systems Target Lung Macrophages with Varying Specificity

To improve our understanding of conditional and conditional inducible systems within the lung and aid in experimental design and interpretation, we assessed the specificity and strength of four different Cre driver lines for targeting macrophages. The two conditional lines selected, LysM-Cre and Csf1r-Cre, are the most highly published “macrophage targeting” lines in the literature (3, 9, 28). The two inducible lines, CX_3_CR1-ERCre and CD68-rtTA, were developed more recently and have been used less extensively. However, based on previous work, we suspected that both lines might be appropriate for targeted studies of IM. Moreover, the two inducible lines have the advantage of strict temporal control. All conditional and conditional inducible lines were crossed with various reporter lines to mark Cre activity, discussed in detail below. We began by assessing reporter expression in the homeostatic lung.

**Figure 1:**
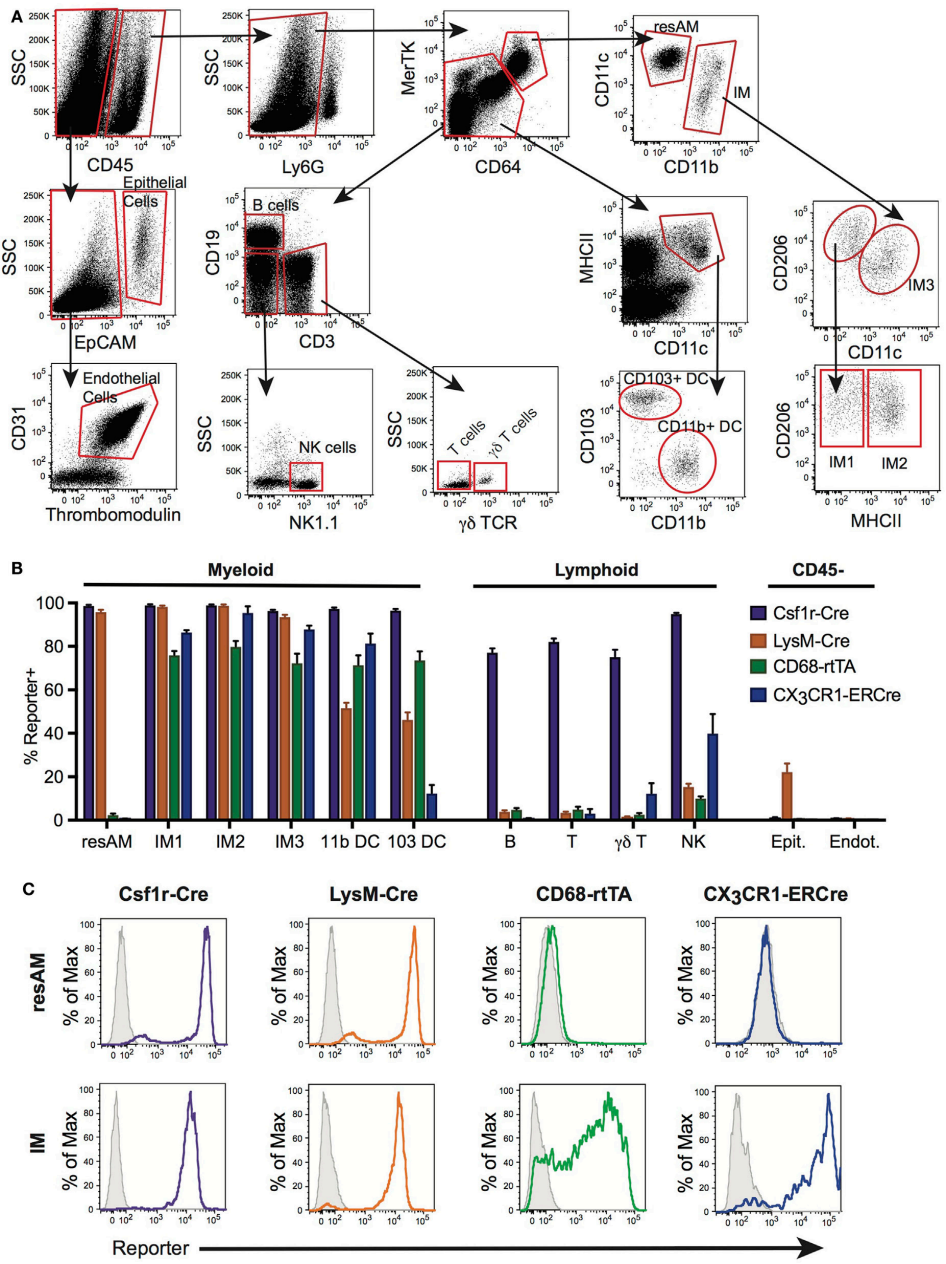
Conditional and inducible promoters vary in lung macrophage targeting. **(A)** Lung digest from reporter mice was assessed by flow cytometry and separated into subpopulations based on surface marker expression. **(B)** Reporter expression in lung subpopulations of myeloid, lymphoid, and structural cells, shown as percent of each population expressing the reporter. **(C)** Representative histograms of reporter expression in resident alveolar macrophages (ResAM) and interstitial macrophages (IM) populations. Data are presented as mean ± SD, *n* = 3–5 mice per group.

Flow cytometry was used to assess reporter expression in cells from digested lung tissue (Figure 1A). Using surface expression of CD45, cells were split into hematopoietic (CD45^+^) and non-hematopoietic (CD45^−^) populations. Within non-hematopoietic cells, we identified epithelial cells as EpCAM^+^, and endothelial cells as EpCAM^−^, CD31^+^ and thrombomodulin^+^. To identify our populations of interest in hematopoietic cells, we first excluded neutrophils using Ly6G. Within Ly6G^−^ cells, macrophages were identified as double-positive for CD64 and MerTK, and non-macrophages as low or negative for CD64 and MerTK expression. From the non-macrophage population, lymphocyte populations were segregated. B cells were identified by their high expression of CD19^+^. CD3^+^ was used to identify T cells that were further identified as γδ T cells or classical αβ T cells by expression of the γδ TCR. Within remaining CD19^−^CD3^−^ cells, NK cells were identified as NK1.1^+^. Also within the non-macrophage population, CD64^−^ and MerTK^−^ cells, DCs were identified by their high expression of MHCII and CD11c, and then further subdivided into CD11b^+^ DC and CD103^+^ DC by expression of their eponymous markers. The macrophage population identified by MerTK and CD64 was divided into CD11c^hi^ ResAM and CD11b^hi^CD11c^hi/lo^ IMs. IMs were then divided into subsets, first using CD11c and CD206 to identify IM3 (CD206^low^), and then remaining cells (CD206^hi^) were defined as IM1 and IM2 using expression of MHCII.

To assess the efficiency and specificity of the Csf1r-Cre and LysM-Cre conditional systems, each strain was crossed with R26-stop^fl/fl^-TdTomato reporter animals. In the resultant offspring, cells that express Cre excise the floxed stop codon upstream of the TdTomato construct and the fluorescent protein is expressed. Both Csf1r-Cre and LysM-Cre were strongly activated in ResAM and IM, with nearly 100% efficiency (Figures 1B,C). However, further examination of Csf1r-Cre demonstrated that this conditional line targeted nearly all leukocytes in the lung, including lymphocytes, with greater than 80% efficiency (Figure 1B). In contrast, LysM-Cre minimally target lymphocytes, although it did target approximately half of Ly6C^hi^ monocytes, CD11b^+^ DC and CD103^+^ DC (Figure 1B) (9). Of significant importance, LysM-Cre targeted nearly a quarter of lung epithelial cells (Figure 1B). Previous studies have suggested that these targeted epithelial cells are alveolar type II cells (1). In comparison, Csf1r-Cre did not target lung epithelium or endothelium. Data presented in Figure 1B are also summarized in Table 1 and representative histograms for all cell populations are shown in Figure S1 in Supplementary Material.

**Table 1:** Reporter expression in cell populations from naïve lung digest and peripheral blood.

		Csf1r-Cre	LysM-Cre	CX_3_CR1-ERCre	CD68-rtTA
Lung digest	ResAM	98.3 ± 0.4	96.1 ± 1.8	≤1	2.59 ± 0.8
	IM1	99.8 ± 0.3	98.5 ± 0.8	86.6 ± 1.5	76.13 ± 3
	IM2	99.6 ± 0.5	98.9 ± 0.8	95.7 ± 5	80 ± 4.3
	IM3	99.8 ± 0.1	93.7 ± 1.9	88.1 ± 2.5	72.5 ± 7.2
	CD11b^+^ DC	99.6 ± 0.4	52.8 ± 3.8	81.6 ± 7.5	71.6 ± 7.3
	CD103^+^ DC	99.7 ± 0.3	44.8 ± 5.5	12.5 ± 6.4	73.8 ± 6.8
	T cells	95.6 ± 6.5	5.2 ± 1.5	3.3 ± 3.2	5.1 ± 1.8
	B cells	79 ± 9.5	5.4 ± 1	≤1	7.4 ± 1
	γδ T cells	95.1 ± 5.2	3.1 ± 0.74	5.4 ± 1.5	2.8 ± 0.8
	NK cells	98.9 ± 1.8	15.6 ± 2.5	40.1 ± 15.2	10.2 ± 1.3
	Endothelial cells	≤1	≤1	≤1	≤1
	Epithelial cells	≤1	22.3 ± 7.3	≤1	≤1

Blood	Neutrophils	99.5 ± 0.4	98 ± 1.1	1.7 ± 1.4	58.7 ± 9.7
	Eosinophils	93 ± 5.1	19.8 ± 1.6	≤1	1.2 ± 0.5
	Ly6C^hi^ monocytes	100 ± 0	67 ± 4.9	51 ± 4.8	70.5 ± 6.3
	Ly6C^lo^ monocytes	99.9 ± 0.9	86.5 ± 1.4	91.5 ± 9.1	63.8 ± 4.3
	T cells	98.5 ± 1	6.7 ± 1.8	6.9 ± 3.3	6.9 ± 2.2
	B cells	82.5 ± 3.5	6.1 ± 0.9	≤1	8.3 ± 2.7

*Data are presented as mean ± SD, n = 3–5 mice per group*.

### Inducible Cre Systems Target Lung Macrophages with Varying Specificity

As a next step, the inducible targeting systems were explored. First, CX_3_CR1-ERCre mice were crossed with R26-stop^fl/fl^-TdTomato reporter mice. In these animals, cells express a mutated Cre that is directed by the CX_3_CR1 promoter and requires tamoxifen as a co-factor for activity. Accordingly, when tamoxifen is administered, *Cre recombinase* becomes active in all CX_3_CR1 expressing cells, thereby excising the floxed R26-stop floxed codon and enabling production of TdTomato. In a similar fashion, CD68-rtTA mice were crossed with tetOn-GFP mice. In these animals, rtTA acts as a transcription factor for the tetOn promoter, but requires the presence of doxycycline for activity. Thus, GFP is expressed in CD68 positive cells following administration of doxycycline. The duration of tamoxifen or doxycycline administration will affect the efficiency of targeting as the percentage of target cells affected increases over time. We have previously reported that CD68-rtTA reporter expression increases to a maximum level over 6 days (29). We therefore assessed CX_3_CR1-ERT2 and CD68-rtTA reporter mice after 1 week of tamoxifen or doxycycline.

CX_3_CR1-ERCre and CD68-rtTA efficiently targeted IM, with 90 and 75% efficiency, respectively (Figures 1B,C). However, ResAM remained reporter negative in both lines, as we have shown previously for CD68-rtTA (29). In addition to IMs, both CX_3_CR1-ERCre and CD68-rtTA targeted lung DCs (Figure 1B). CX_3_CR1-ERCre also targeted a significant portion of NK cells, although targeting of other lymphocytes remained low (Figure 1B). Reporter expression was not observed in endothelial or epithelial cells for either line. The data presented in Figure 1B is also summarized in Table 1 and representative histograms for all cell populations are shown in Figure S1 in Supplementary Material.

### Conditional and Inducible Cre Systems Target Blood Myeloid Cells with Varying Specificity

**Figure 2:**
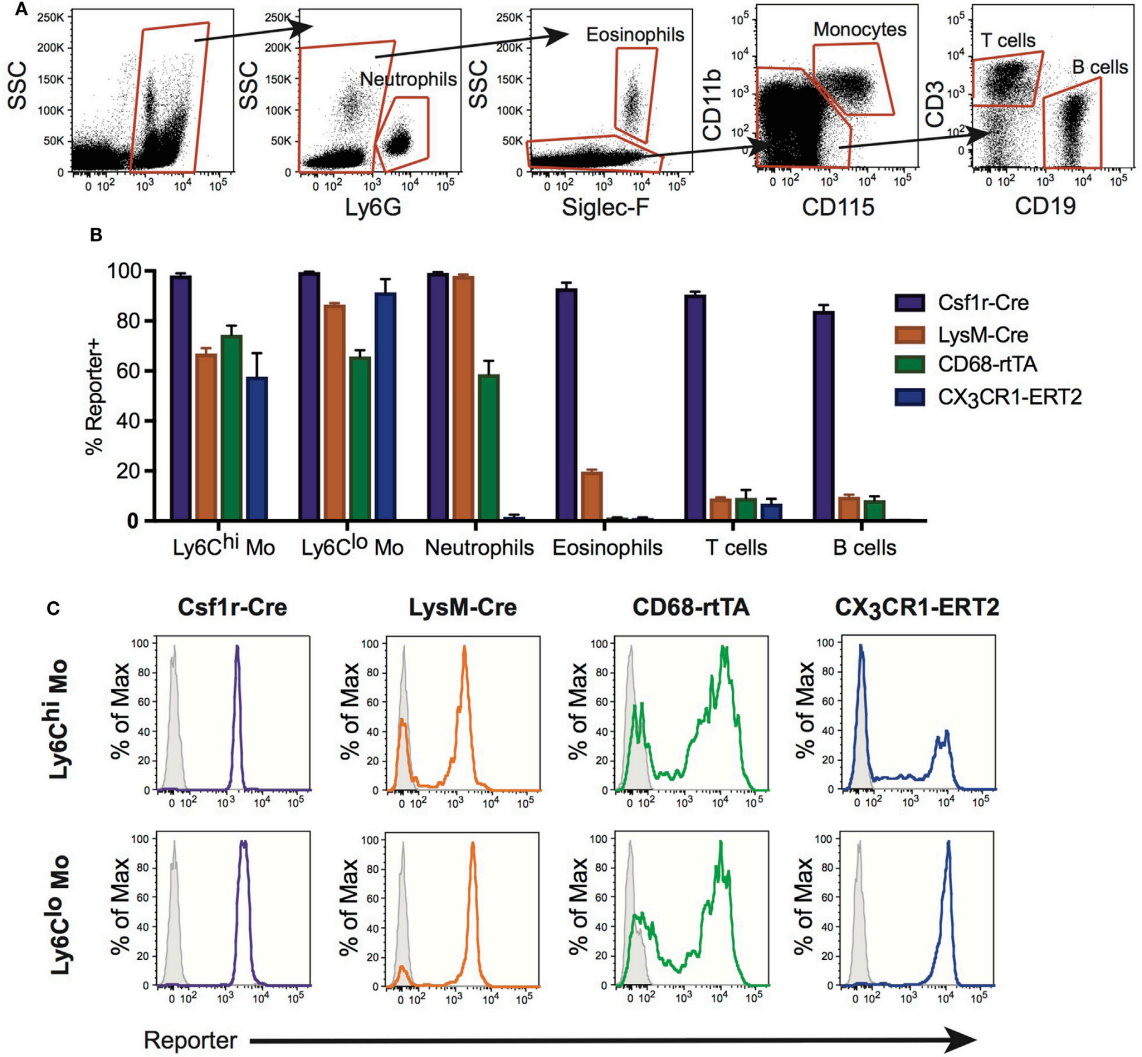
Conditional and inducible promoters vary in blood myeloid targeting. **(A)** Peripheral blood from reporter mice was assessed by flow cytometry and separated into subpopulations based on surface marker expression. **(B)** Reporter expression in blood subpopulations, shown as percent of each population expressing the reporter. **(C)** Representative histograms of reporter expression in Ly6C^hi^ and Ly6C^lo^ monocyte populations. Data are presented as mean ± SD, *n* = 3–5 mice per group.

During homeostasis, Ly6C^hi^ monocytes constitutively traffic through the lungs (30). During inflammation, emigration of these cells from the circulation is increased and it is believed that they give rise to RecMΦ. Neutrophils and other leukocytes also emigrate from the bloodstream during inflammation. Therefore, we assessed activation of Csf1r-Cre, LysM-Cre, CX_3_CR1-ERCre, and CD68-rtTA in blood leukocytes. Blood was obtained by cardiac puncture, red cells were lysed, and reporter expression was measured by flow cytometry (Figure 2A). First, CD45^+^ leukocytes were identified. Neutrophils were identified using Ly6G, and eosinophils distinguished using Siglec-F. Monocytes were identified from remaining cells as CD115^+^CD11b^+^. B and T cells were identified within the remaining pool as CD19^+^ or CD3^+^, respectively.

Ly6C^hi^ and Ly6C^lo^ monocyte subsets were targeted by all four lines, with the highest percentage of cells targeted by Csf1r-Cre (Figures 2B,C). Csf1r-Cre, LysM-Cre, and CD68-rtTA were activated in neutrophils, and both Csf1r-Cre and LysM-Cre were activated in eosinophils (Figure 2B). Only Csf1r-Cre was completely activated in lymphocytes (Figure 2B). The data presented in Figure 2B is also summarized in Table 1 and representative histograms for all cell populations are shown in Figure S2 in Supplementary Material.

Although resident tissue macrophages of other organs do not directly migrate to the lung, macrophages of other organs, particularly the bone marrow and spleen, regulate hematopoiesis, and impact circulating leukocyte levels (31–33). We examined reporter expression of Csf1r-Cre, LysM-Cre, CX_3_CR1-ERCre, and CD68-rtTA-driven lines in bone marrow, spleen, and PL using flow cytometry (Figures S3A,B,E in Supplementary Material). Macrophages in all three tissues were targeted by all four drivers (Figures S3C,D,F,G in Supplementary Material).

### Timing of Inducible Cre Systems Can Be Used to Refine Specificity for Lung IM

A distinct advantage of inducible Cre systems such as CX_3_CR1-ERCre and CD68-rtTA is that the timing and duration of tamoxifen or doxycycline administration can be used to enhance cell selectivity and specificity. As an example, we explored the kinetics of dosing in both inducible strains to refine targeting of IM. Mice were treated with either tamoxifen or doxycycline for 1 week, then the drug was withdrawn and reporter expression was examined 1, 2, and 4 weeks later.

**Figure 3:**
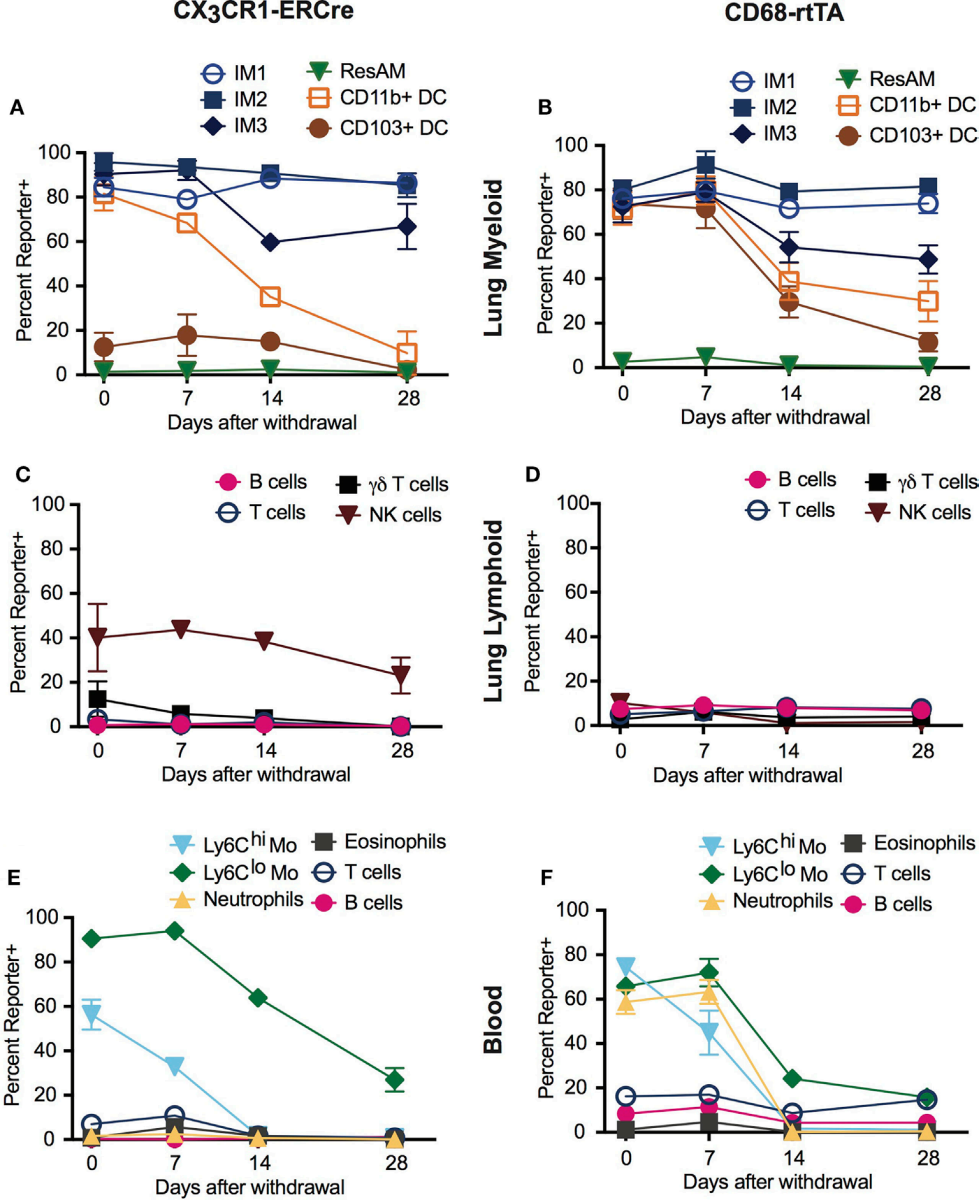
Dosing of tamoxifen and doxycycline can be manipulated to enhance targeting of interstitial macrophages (IMs) in inducible systems. CX_3_CR1-estrogen receptor-Cre (ERCre) and CD68-rtTA reporter mice were administered tamoxifen or doxycycline, respectively, for 1 week, then administration was halted and mice were examined 1, 2, and 4 weeks later. **(A)** Reporter expression in lung myeloid cells from CX_3_CR1-ERCre mice over time after withdrawal from tamoxifen. **(B)** Reporter expression in lung myeloid cells from CD68-rtTA mice over time after withdrawal from doxycycline. **(C)** Reporter expression in lung lymphoid cells from CX_3_CR1-ERCre mice over time after withdrawal from tamoxifen. **(D)** Reporter expression in lung lymphoid cells from CD68-rtTA mice over time after withdrawal from doxycycline. **(E)** Reporter expression in peripheral blood cells from CX_3_CR1-ERCre mice over time after withdrawal from tamoxifen. **(F)** Reporter expression peripheral blood cells from CD68-rtTA mice over time after withdrawal from doxycycline. Data are presented as mean ± SD, *n* = 3–5 mice per group.

In the lung tissue of both CX_3_CR1-ERCre and CD68-rtTA mice, IM1 and IM2 reporter expression remained stable after tamoxifen or doxycycline was withdrawn, with only a 5% decrease in reporter positive cells over 4 weeks (Figures 3A,B). IM3 showed a steeper rate of declining reporter expression, with a 20% decrease in reporter positive cells (Figures 3A,B). As anticipated, ResAM were reporter negative at all times. Importantly, the percentage of reporter positive DC dropped to less than 10% in CX_3_CR1-ERCre mice by 4 weeks and to less than 30% in CD68-rtTA mice. Reporter expression was similarly low in lymphocytes isolated from the lungs, with the exception of NK cells in CX_3_CR1-ERCre mice, in which nearly 25% remained positive (Figures 3C,D).

Reporter expression in neutrophils and monocytes from peripheral blood was also lost after 4 weeks of tamoxifen or doxycycline withdrawal (Figures 3E,F). Virtually, all of the neutrophils and Ly6C^hi^ monocytes were TdTomato reporter negative, whereas approximately 20% of Ly6C^lo^ monocytes remained TdTomato positive in both strains. These findings support the concept that neutrophils and Ly6C^hi^ monocytes have relatively short lifespans, and that turnover of neutrophils and Ly6C^hi^ monocytes are relatively high (30). In addition, since excision of floxed alleles is permanent in affected cells (i.e., cells stay reporter positive for life), the observation that the IM3 population gradually loses reporter expression suggests that at least a portion of these cells are replaced by monocyte precursors, supporting previous reports using parabiotic mice (14). Taken together, these findings show that dosing of tamoxifen and doxycycline can be manipulated to enhance targeting of IMs in CD68-rtTA and CX_3_CR1-ERCre mice.

### Conditional Cre Systems Target Recruited Alveolar Macrophages following LPS Injury

**Figure 4:**
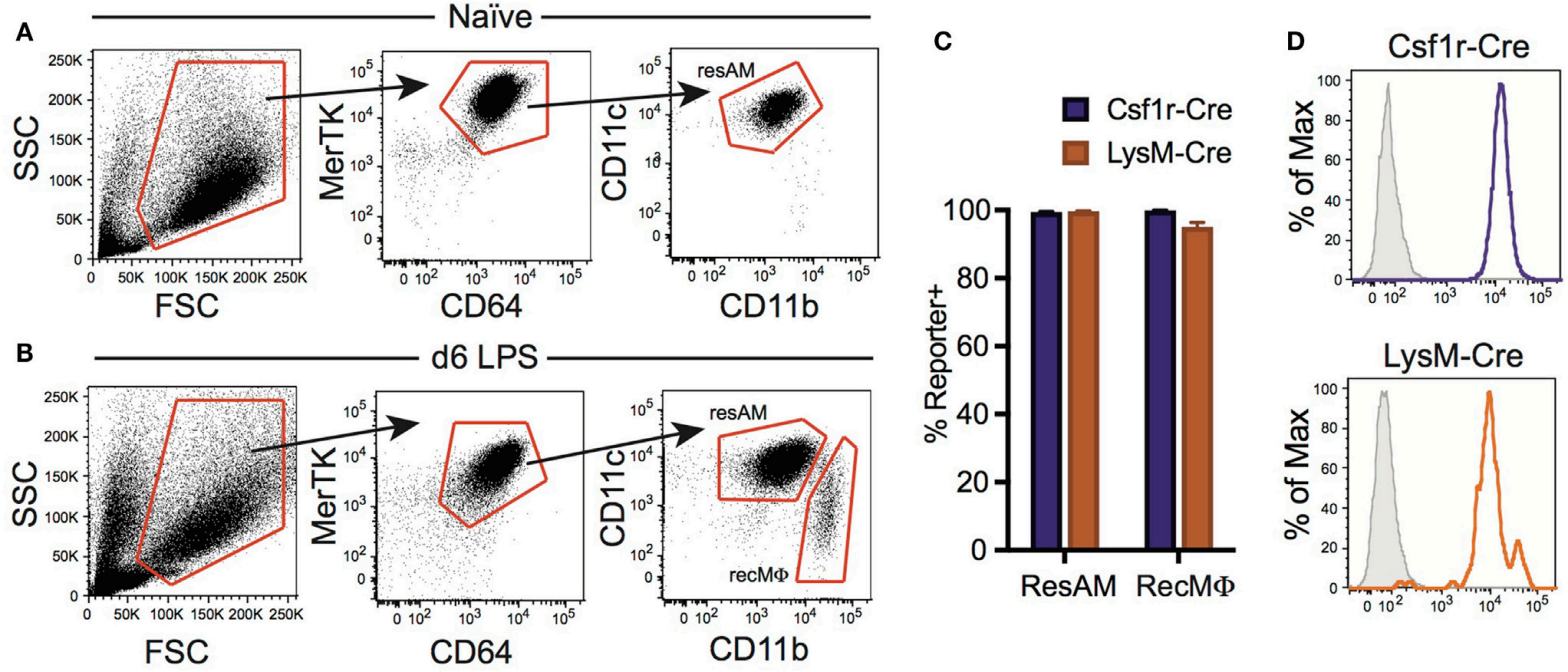
Csf1r-Cre and LysM-Cre promoters target RecMΦ after LPS lung injury. Csf1r-Cre and LysM-Cre reporter mice were administered intratracheally (IT) LPS. After 6 days, reporter expression was examined. **(A)** Bronchoalveolar lavage (BAL) from naïve mice contains only CD11c^hi^CD11b^lo^ resident alveolar macrophages (ResAM). **(B)** BAL from mice 6 days after IT LPS contains ResAM and RecMΦ subpopulations that can be separated based on CD11b and CD11c expression. **(C)** Reporter expression in ResAM and RecMΦ after LPS. **(D)** Representative histograms of RecMΦ reporter expression. Data are presented as mean ± SD, *n* = 4 mice per group.

Inflammation has been reported to alter Cre activity, and during inflammation a new population of macrophages is present in the lung airways: RecMΦ. This macrophage population is believed to arise from infiltrating monocytes maturing *in situ*, and can be distinguished from ResAM by high expression of CD11b (Figure 4A) [and low expression of Siglec-F (17, 34)]. We assessed Csf1r-Cre and LysM-Cre reporter mice following LPS injury for the potential changes to Cre activity and measured reporter expression in recruited macrophages. RecMΦ were targeted by both Csf1r-Cre and LysM-Cre with near 100% efficiency (Figures 4B–D). Efficiency for all three IM populations was also high (>95%; Table 2). However, as seen during homeostasis, other leukocyte populations were also highly targeted in Csf1r-Cre mice and to a lesser extent in LysM-Cre animals (Table 2).

**Table 2:** Reporter expression in cell populations from bronchoalveolar lavage (BAL), lung digest, and peripheral blood 6 days after intratracheally LPS.

		Csf1r-Cre	LysM-Cre
BAL	ResAM	98.9 ± 0.7	96.6 ± 0.9
	RecMΦ	99.9 ± 0.2	95.2 ± 2.5

Lung digest	IM1	96.6 ± 0.7	90.2 ± 2.7
	IM2	98.2 ± 0.8	93.9 ± 0.5
	IM3	98.9 ± 0.6	93.5 ± 0.9
	CD11b^+^ DC	98.6 ± 0.9	53.6 ± 4.4
	CD103^+^ DC	98.4 ± 0.6	48.4 ± 6.8
	T cells	91.9 ± 1.4	3.1 ± 0.7
	B cells	79.9 ± 6.8	5.2 ± 0.8
	γδ T cells	91.5 ± 2.3	3.4 ± 1.1
	NK cells	97.6 ± 0.5	14.7 ± 1.1

Blood	Neutrophils	99.8 ± 0.2	98.9 ± 0.5
	Eosinophils	98.8 ± 0.4	12.5 ± 0.5
	Ly6C^hi^ monocytes	99.9 ± 0.3	78.2 ± 1.7
	Ly6C^lo^ monocytes	99.9 ± 0.2	88.1 ± 1.0
	T cells	99.8 ± 0.1	4.2 ± 0.8
	B cells	87.8 ± 4.2	2.9 ± 0.4

*Data are presented as mean ± SD, n = 4 mice per group*.

### ResAM Are Resistant to rtTA-Driven Doxycycline-Induced Transgene Expression

**Figure 5:**
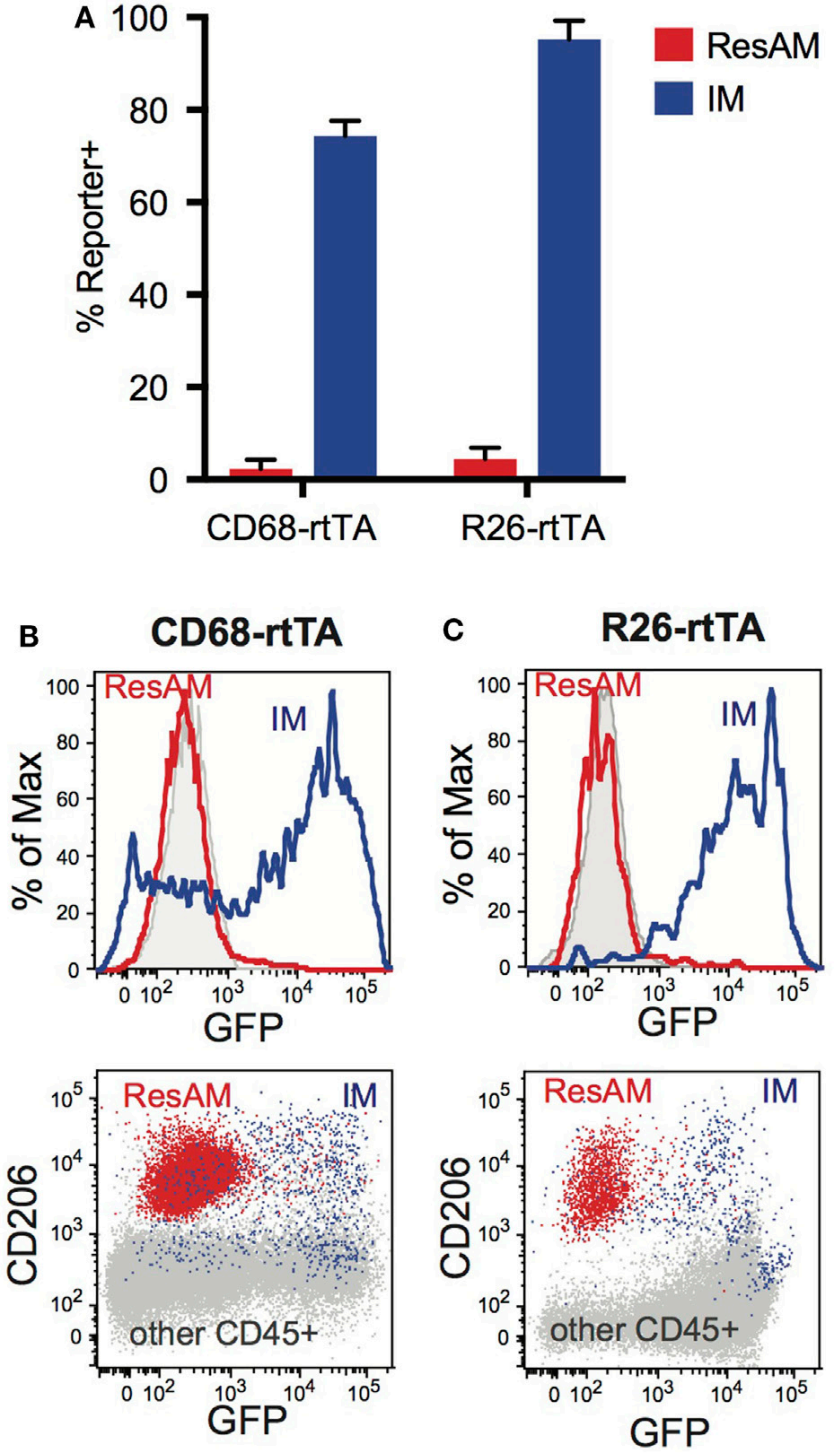
Expression of rtTA and administration of doxycycline fails to activate tet-On genes in resident alveolar macrophages (ResAM). CD68-rtTA and R26-rtTA reporter mice were administered doxycycline for 10 months, then reporter expression was examined in the lung. **(A)** Reporter expression in ResAM and interstitial macrophages (IM) after 10 months of doxycycline administration. **(B)** Representative histograms of ResAM (red) and IM (blue) from CD68-rtTA reporter mice overlaid on ResAM from non-reporter control animals (gray); dot plot of ResAM (red) and IM (blue) from CD68-rtTA reporter mice overlaid on all other CD45^+^ cells (gray) from R26-rtTA reporter mice. **(C)** Representative histograms of ResAM (red) and IM (blue) from R26-rtTA reporter mice overlaid on ResAM from non-reporter control animals (gray); dot plot of ResAM (red) and IM (blue) from R26-rtTA reporter mice overlaid on all other CD45^+^ cells (gray) from R26-rtTA reporter mice. Data are presented as mean ± SD, *n* = 4–5 mice per group.

We have previously reported that ResAM do not activate CD68-rtTA *in vivo* after 21 days of doxycycline, or *in vitro* when doxycycline was provided in culture media for 4 days (29). However, it was unclear whether this restriction reflected a failure to activate the CD68 promoter or resulted from decreased rtTA activity. To assess this, we obtained R26-rtTA mice (35), where rtTA is expressed under control of the Rosa26 promoter, which is ubiquitously expressed by all leukocytes including ResAM (36). Both CD68-rtTA and R26-rtTA mice were crossed with tetOn-GFP mice, and resulting offspring were administered doxycycline for 10 months. Notably, ResAM from CD68-rtTA mice remained GFP negative, although the majority of myeloid cells (including IM) were GFP positive (Figures 5A,B). Strikingly, ResAM from R26-rtTA mice also failed to express GFP, whereas >95% of other leukocytes were GFP positive (Figures 5A,C). We conclude that ResAM are resistant to the activation of rtTA-driven systems. Accordingly, the CD68-rtTA system can be exploited to target-specific lung tissue macrophage populations and to distinguish the roles they play during both homeostasis and in lung disease.

## Discussion

Cre-LoxP strategies are commonly employed to delete genes in specific cell types or tissues. However, accurate data interpretation requires a firm understanding of the underlying genetic principles and detailed knowledge regarding the specificity and efficiency of the Cre driver. To this end, we assessed the operating characteristics of four commonly used and commercially available mouse lines that are published as “macrophage targeting”: Csf1r-Cre, LysM-Cre, CX_3_CR1-ERCre, and CD68-rtTA. We found that each line targeted multiple non-macrophage populations in the lungs and circulation. In conditional deletion systems, LysM-Cre had greater specificity for lung macrophages than Csf1r-Cre but also targeted type II alveolar cells, and was less efficient at targeting monocytes. The CX_3_CR1-ERCre and CD68-rtTA systems robustly targeted IMs and trafficking monocytes, but did not delete floxed genes in resident AMs. Non-macrophage populations were also targeted in these inducible systems; however, macrophage specificity was improved once tamoxifen or doxycycline was withdrawn. As discussed below, these findings can be used to one’s advantage and can enable time and cell-specific targeting of specific lung macrophage populations.

It is critical to appreciate that in conditional and inducible Cre lines, once a cell activates the driver promoter, floxed DNA sequences will be permanently deleted from that time forward—even if the driver promoter is later downregulated. Moreover, the floxed sequences will also be permanently deleted in all daughter cells. This point can be best illustrated by comparing CX_3_CR1-Cre mice (11) (not studied herein) to CX_3_CR1-ERCre mice. In conditional CX_3_CR1-Cre mice, floxed alleles are deleted in ResAM because the precursors for ResAM express CX_3_CR1 during embryogenesis. However, in the inducible CX_3_CR1-ERCre mice ResAM are not targeted when tamoxifen is administered to adult mice because mature ResAM do not express CX_3_CR1 and are not replaced by circulating monocytes (11).

Importantly, the cell specificity of Cre-LoxP strategies may differ from that of non-Cre reporter systems. For instance, in our studies the Csf1r-Cre system affects almost all leukocytes. In comparison, when the Csf1r promoter is used to directly drive a fluorescent reporter [as in the Mafia mouse (37)], the fluorescent protein is only expressed when the Csf1r promoter is actively engaged. When promoter engagement ceases, GFP only remains until it is degraded, or until the cell undergoes sufficient divisions to dilute the signal. Accordingly, labeling is primarily restricted to myeloid cells in the Mafia mouse, even though the promoter is the same as in Csf1r-Cre animals. We speculate that early, irreversible recombination in hematopoietic precursor cells is responsible for the non-specific Cre activation in Csf1r-Cre mice even though in mature leukocytes Csf1r expression is restricted to myeloid cells.

The four Cre driver lines that we tested each have positives and negatives for their use. Csf1r-Cre targets ResAM and IM with the highest efficiency, but the lowest specificity. All leukocytes examined were targeted by Csf1r-Cre, although Csf1r-Cre did not target lung epithelium or endothelium. In contrast, nearly a quarter of lung epithelium was targeted by LysM-Cre. ResAM and IM were also targeted with high efficiency by LysM-Cre, but neutrophils, DC, and monocytes were affected as well. CX_3_CR1-ERCre targeted IM with high efficiency but did not target ResAM. CX_3_CR1-ERCre also targeted CD11b^+^ DC, Ly6C^lo^ monocytes, and NK cells, but did not affect other cell populations including lung endothelial and epithelial cells. Off-target effects on non-macrophage populations decreased once tamoxifen was withdrawn as CD11b^+^ DC, monocytes, and NK cells were replaced by unaffected precursors. CD68-rtTA targeted IM with moderate efficiency, and did not target ResAM. CD68-rtTA also targeted DC, monocytes, and neutrophils, but did not affect lymphocytes, endothelial, or epithelial cells. As seen with CX_3_CR1-ERCre, off-target effects on non-macrophage populations decreased once doxycycline was withdrawn as DC, monocytes, and neutrophils were replaced by unaffected precursors.

Inducible CX_3_CR1-ERCre and CD68-rtTA mice provide an opportunity to discretely target IM without affecting ResAM. IM have been far less studied then ResAM due to the difficult nature of their isolation and their low numbers compared with ResAM. Hence, since the inducible model systems described herein uniquely target IM, these provide a critical advance for their study. Notably, the CX_3_CR1-ERCre system targets IM with slightly greater efficiency than CD68-rtTA and targets fewer DC, and no neutrophils, suggesting this may be the superior model. However, CX_3_CR1-ERCre affects NK cells, which is not observed in CD68-rtTA mice. Accordingly, both models represent powerful tools for the study of lung IM, especially when deletion of floxed alleles in ResAM is not desirable.

While the biologic mechanism that restricts CX_3_CR1-ERCre targeting in ResAM is clear (as discussed above), the failure of CD68-rtTA to target ResAM is less apparent since mature ResAM express high levels of CD68 (14, 29). To this end, we examined activation of the R26-rtTA line in ResAM as a comparator. Like CD68, endogenous R26 protein is expressed by mature ResAM. However, R26-rtTA was not activated in ResAM. We therefore suggest that ResAM may not be amenable to targeting with doxycycline-inducible systems. Although not tested here, we speculate that ResAM have the ability to export doxycycline from the cell, thereby preventing its action as a co-factor in the nucleus. Notably, ResAM uniquely express high levels of ABCG1, a homolog for bacterial transporters of tetracyclines. This does not diminish the utility of CD68-rtTA for lung studies, but should caution investigators about attempting to use any rtTA system to target ResAM.

Although we have identified two viable lines for the study of IM, none of the lines examined in this paper specifically target ResAM. CD11c-Cre has been reported to target ResAM, although careful studies of its specificity and efficiency have not been published. Since CD11c is expressed by DC, trafficking monocytes in tissue, some IM, and some lymphocytes (particularly NK cells), we expect that its targeting would be non-specific. Mafb-Cre provides an alternative possibility for targeting ResAM because it is required for the differentiation of monocytes and macrophages, but suppresses DC and granulocyte differentiation (38, 39). Although *Mafb* expression in mature ResAM is very low, ResAM are targeted by Mafb-Cre (14, 39) presumably due to activation in precursors. Additionally, Mafb-Cre minimally affects DC or granulocytes, and although bulk monocytes express Mafb, reporter mice showed that Mafb-Cre targeted only 10% of Ly6C^hi^ and 40% of Ly6C^lo^ monocytes (39). RecMΦ in the lung following exposure to house dust mite antigen are targeted by Mafb-Cre (39). IM targeting has not been assessed, although we would expect IM to be efficiently targeted. Targeting of B cells was found to be very low (39), although targeting of other lymphocytes or lung epithelial cells was not reported. The lack of DC and granulocyte targeting makes Mafb-Cre a promising line for discrete targeting of macrophages. Both CD11c-Cre and Mafb-Cre are commercially available. However, both CD11c-Cre and Mafb-Cre will target macrophages in many organs, not specifically lung macrophages, as we observed for Csf1r-Cre, LysM-Cre, CX_3_CR1-ERCre, and CD68-rtTA. In addition, Mafb-Cre will not discriminate gene deletion between ResAM and IM in the lung. Lack of organ specificity is another limiting factor of “macrophage specific” conditional and inducible systems. We hypothesize a tamoxifen-inducible Siglec-F-ERCre might overcome this limitation and provide a lung ResAM-specific inducible system. ResAM are the only tissue macrophage known to express Siglec-F (34, 40). Siglec-F is not expressed by DC or monocytes, although it is expressed by granulocytes, particularly eosinophils. Theoretically, a pulse-wait of tamoxifen should target ResAM while targeted granulocytes turnover and are replaced from untargeted precursors. To our knowledge, no Siglec-F-driven lines have been developed.

The use of animal models remains a powerful tool for understanding the homeostatic functions of lung macrophages and their roles in pulmonary disease. While useful, Cre-LoxP systems are imperfect, and understanding their limitations is necessary for effective use. With a firm understanding of the populations targeted by each driver, vagaries of the system may even be harnessed to one’s advantage, such as in the case of CD68-rtTA mice being used to target IM without targeting ResAM. In general, neither Csf1r-Cre nor LysM-Cre are specific for macrophages—a problem for their use in the study of lung macrophages. Inducible CX_3_CR1-ERCre and CD68-rtTA systems show increased specificity for macrophages, particularly after pulse-wait of tamoxifen or doxycycline, however, they only target IM and not ResAM. Ultimately, each investigator must choose for themselves which system introduces the fewest confounding factors for their research. The data provided herein aim to assist with that decision, but there is clear room for improvement in developing new lines for conditional targeting of lung macrophages.

## Ethics Statement

This study was carried out in accordance with the recommendations of the Institutional Animal Care and Use Committee at National Jewish Health. The protocol was approved by the Institutional Animal Care and Use Committee at National Jewish Health (approval #AS2574-01-20).

## Author Contributions

AM, WJ, and CJ conceived of the study; AM and KA executed experiments; AM, WJ, CJ, and AL-S prepared the manuscript; all authors provided intellectual input, critical feedback, discussed results, and designed experiments.

## Conflict of Interest Statement

The authors declare that the research was conducted in the absence of any commercial or financial relationships that could be construed as a potential conflict of interest.

## Supplementary Material

Figure S1 Representative histograms of reporter expression in lung cell populations from reporter lines. For each lung cell population assessed in Figure 1, representative histograms of reporter expression are shown from Csf1r-Cre, LysM-Cre, CX_3_CR1-estrogen receptor-Cre (ERCre), and CD68-rtTA reporter mice. CX_3_CR1-ERCre and CD68-rtTA data are from mice administered tamoxifen or doxycycline for 1 week. Gray histogram shows florescence of cells from non-reporter control mice.

Figure S2 Representative histograms of reporter expression in peripheral blood populations from reporter lines. For each peripheral blood population assessed in Figure 2, representative histograms of reporter expression are shown from Csf1r-Cre, LysM-Cre, CX_3_CR1-estrogen receptor-Cre (ERCre), and CD68-rtTA reporter mice. CX_3_CR1-ERCre and CD68-rtTA data are from mice administered tamoxifen or doxycycline for 1 week. Gray histogram shows florescence of cells from non-reporter control mice.

Figure S3 Conditional and inducible promoters vary in targeting bone marrow, splenic, and peritoneal macrophages. **(A)** Spleen from reporter mice was assessed by flow cytometry and separated into subpopulations based on surface marker expression. **(B)** Bone marrow from reporter mice was assessed by flow cytometry and separated into subpopulations based on surface marker expression. **(C)** Reporter expression in splenic and bone marrow CD11b^+^CD11c^+^Gr-1^−^ macrophages, shown as percent of cells expressing the reporter. **(D)** Representative histograms of reporter expression of CD11b^+^CD11c^+^Gr-1^−^ splenic and bone marrow macrophages. **(E)** Peritoneal lavage from reporter mice was assessed by flow cytometry and separated into subpopulations based on surface marker expression. **(F)** Reporter expression of Tim4^hi^ and Tim4^lo^ peritoneal macrophages, shown as percent of cells expressing the reporter. **(G)** Representative histograms of reporter expression in peritoneal macrophages. Resident peritoneal macrophages were not observed in lavage following IP tamoxifen, therefore no data are shown for peritoneal macrophages from CX_3_CR1-estrogen receptor-Cre mice. Data are presented as mean ± SD, *n* = 3–5 mice per group.
