# Supplementary figures and images for: Promoter Specificity and Efficacy in Conditional and Inducible Transgenic Targeting of Lung Macrophages
Source: Front Immunol. 2017 Nov 24;8:1618. doi: 10.3389/fimmu.2017.01618 (PMC5705560; doi:10.3389/fimmu.2017.01618)

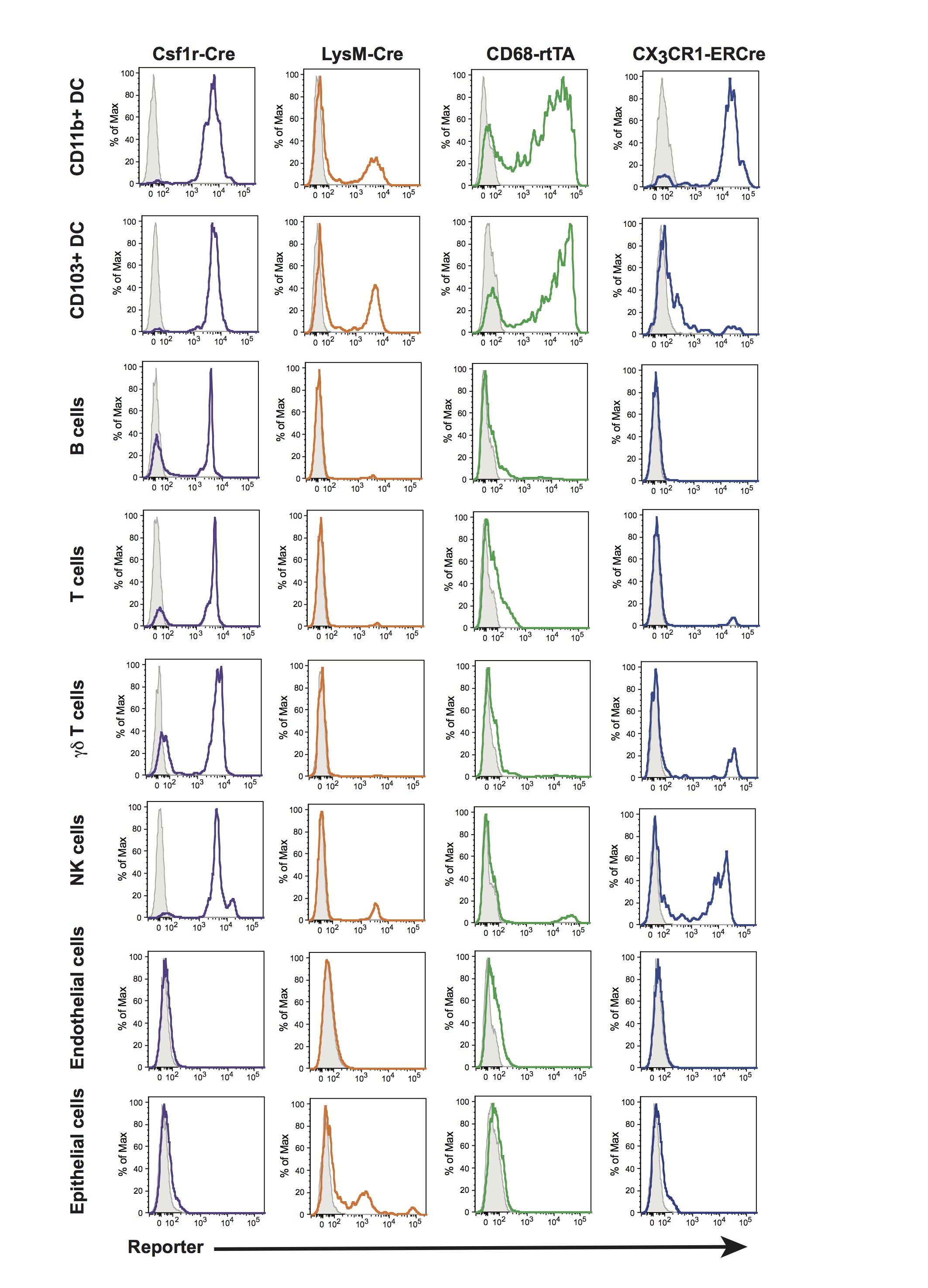

Supplement: Figure S1 — Representative histograms of reporter expression in lung cell populations from reporter lines. For each lung cell population assessed in Figure 1, representative histograms of reporter expression are shown from Csf1r-Cre, LysM-Cre, CX3CR1-estrogen receptor-Cre (ERCre), and CD68-rtTA reporter mice. CX3CR1-ERCre and CD68-rtTA data are from mice administered tamoxifen or doxycycline for 1 week. Gray histogram shows florescence of cells from non-reporter control mice. [file image_1.jpeg]

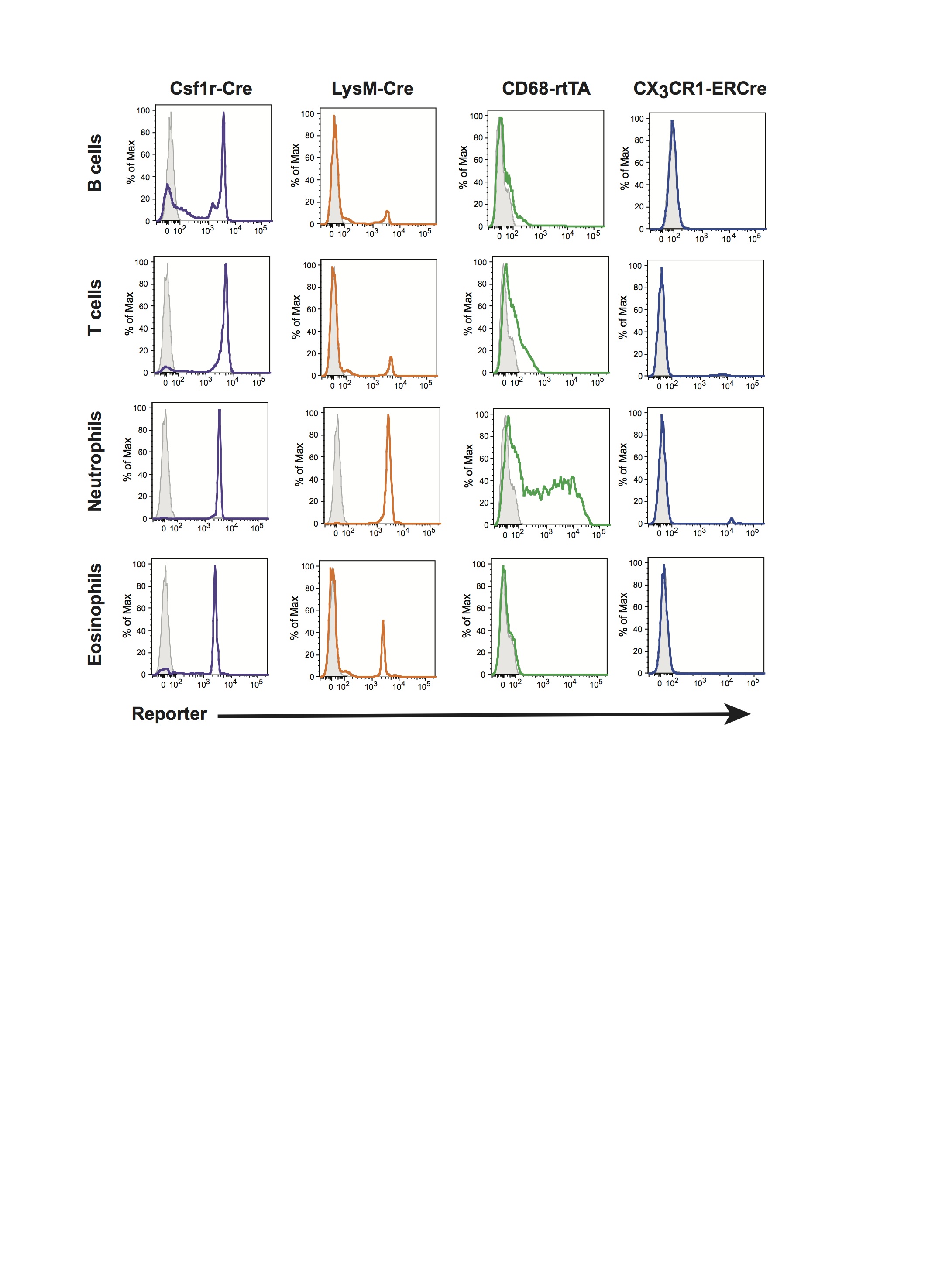

Supplement: Figure S2 — Representative histograms of reporter expression in peripheral blood populations from reporter lines. For each peripheral blood population assessed in Figure 2, representative histograms of reporter expression are shown from Csf1r-Cre, LysM-Cre, CX3CR1-estrogen receptor-Cre (ERCre), and CD68-rtTA reporter mice. CX3CR1-ERCre and CD68-rtTA data are from mice administered tamoxifen or doxycycline for 1 week. Gray histogram shows florescence of cells from non-reporter control mice. [file image_2.jpeg]

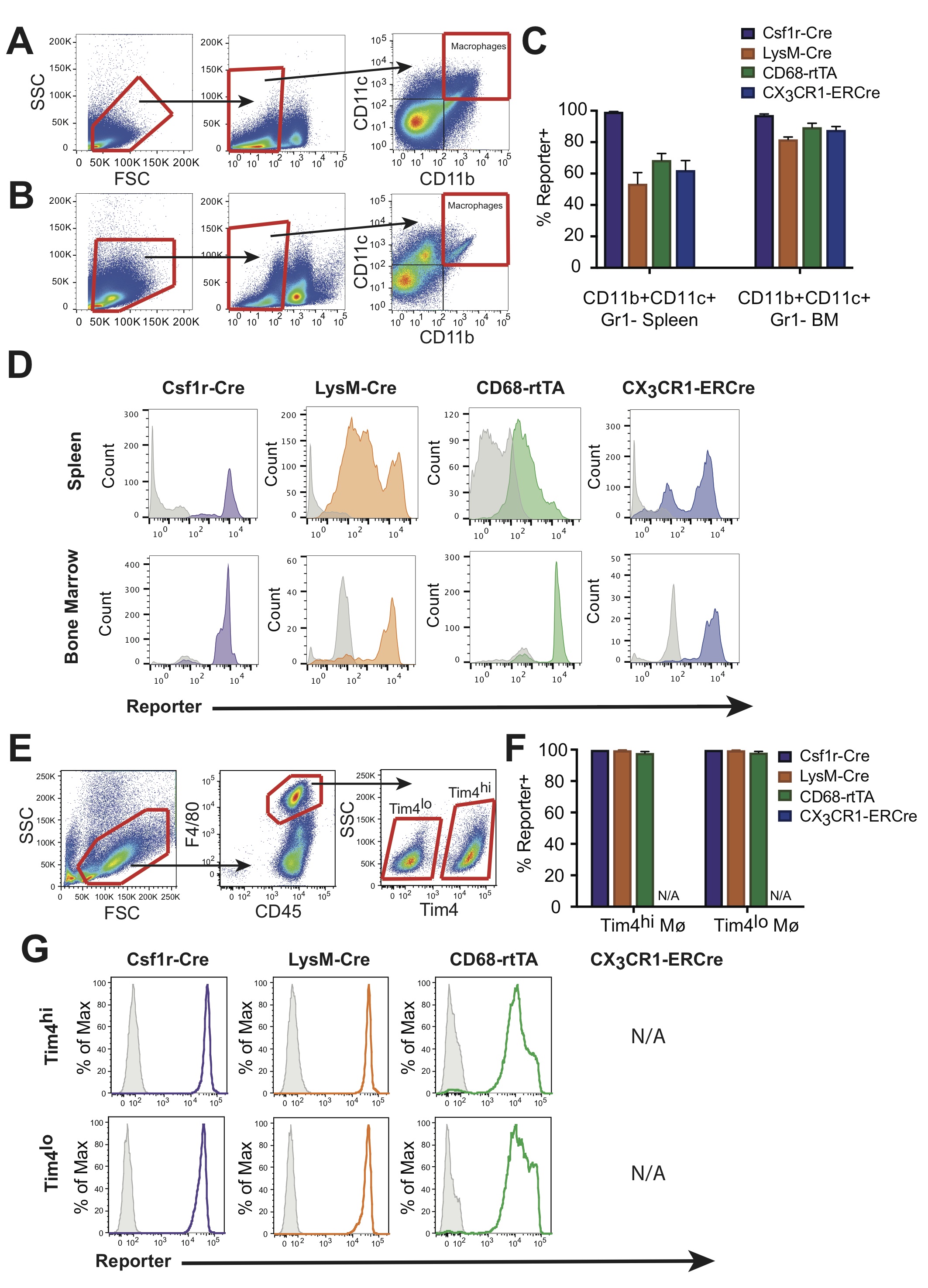

Supplement: Figure S3 — Conditional and inducible promoters vary in targeting bone marrow, splenic, and peritoneal macrophages. (A) Spleen from reporter mice was assessed by flow cytometry and separated into subpopulations based on surface marker expression. (B) Bone marrow from reporter mice was assessed by flow cytometry and separated into subpopulations based on surface marker expression. (C) Reporter expression in splenic and bone marrow CD11b+CD11c+Gr-1− macrophages, shown as percent of cells expressing the reporter. (D) Representative histograms of reporter expression of CD11b+CD11c+Gr-1− splenic and bone marrow macrophages. (E) Peritoneal lavage from reporter mice was assessed by flow cytometry and separated into subpopulations based on surface marker expression. (F) Reporter expression of Tim4hi and Tim4lo peritoneal macrophages, shown as percent of cells expressing the reporter. (G) Representative histograms of reporter expression in peritoneal macrophages. Resident peritoneal macrophages were not observed in lavage following IP tamoxifen, therefore no data are shown for peritoneal macrophages from CX3CR1-estrogen receptor-Cre mice. Data are presented as mean ± SD, n = 3–5 mice per group. [file image_3.jpeg]
